# DCAF1-targeting microRNA-3175 activates Nrf2 signaling and inhibits dexamethasone-induced oxidative injury in human osteoblasts

**DOI:** 10.1038/s41419-021-04300-8

**Published:** 2021-10-29

**Authors:** Jing Chen, Jin-qian Liang, Yun-Fang Zhen, Lei Chang, Zhen-tao Zhou, Xiong-jie Shen

**Affiliations:** 1 Department of Endocrinology, Hunan Provincial People’s Hospital, The First Affiliated Hospital of Hunan Normal University, Changsha, China; 2 Department of Orthopaedics, Peking Union Medical College Hospital, Beijing, China; 3 The Center of Diagnosis and Treatment for Children’s Bone Diseases, The Children’s Hospital of Soochow University, Suzhou, China; 4 Department of Spine Surgery, Hunan Provincial People’s Hospital, The First Affiliated Hospital of Hunan Normal University, Changsha, China; 5 Department of Orthopedics, The Second Affiliated Hospital of Soochow University, Suzhou, China

**Keywords:** Apoptosis, Stress signalling

## Abstract

Activation of nuclear-factor-E2-related factor 2 (Nrf2) signaling can protect human osteoblasts from dexamethasone-induced oxidative injury. DDB1 and CUL4 associated factor 1 (DCAF1) is a novel ubiquitin E3 ligase for Nrf2 protein degradation. We identified a novel DCAF1-targeting miRNA, miR-3175. RNA pull-down, Argonaute 2 RNA-immunoprecipitation, and RNA fluorescent in situ hybridization results confirmed a direct binding between miR-3175 and *DCAF1* mRNA in primary human osteoblasts. *DCAF1* 3′-untranslated region luciferase activity and its expression were significantly decreased after miR-3175 overexpression but were augmented with miR-3175 inhibition in human osteoblasts and hFOB1.19 osteoblastic cells. miR-3175 overexpression activated Nrf2 signaling, causing Nrf2 protein stabilization, antioxidant response (ARE) activity increase, and transcription activation of Nrf2-dependent genes in human osteoblasts and hFOB1.19 cells. Furthermore, dexamethasone-induced oxidative injury and apoptosis were largely attenuated by miR-3175 overexpression in human osteoblasts and hFOB1.19 cells. Importantly, shRNA-induced silencing or CRISPR/Cas9-mediated Nrf2 knockout abolished miR-3175 overexpression-induced osteoblast cytoprotection against dexamethasone. Conversely, DFAC1 knockout, by the CRISPR/Cas9 method, activated the Nrf2 cascade and inhibited dexamethasone-induced cytotoxicity in hFOB1.19 cells. Importantly, miR-3175 expression was decreased in necrotic femoral head tissues of dexamethasone-taking patients, where *DCAF1* mRNA was upregulated. Together, silencing DCAF1 by miR-3175 activated Nrf2 signaling to inhibit dexamethasone-induced oxidative injury and apoptosis in human osteoblasts.

## Introduction

Dexamethasone (DEX) is commonly utilized in patients suffering from inflammatory and autoimmune diseases [1]. Yet, long-term or high-dose use of DEX would lead to direct cytotoxicity to osteoblasts, serving as one main cause of osteoporosis and/or osteonecrosis [2, 3]. Our group and others have been exploring the underlying pathological mechanisms of DEX-caused osteoblast injury [4–9].

In the unstimulated condition, nuclear-factor-E2-related factor 2 (Nrf2) is mainly inactivated in the cytoplasm, directly interacting with its suppressor protein Kelch-like ECH-associated protein 1 (Keap1). The latter mediates ubiquitination and proteasomal degradation of the Nrf2 protein [10–12]. With different stimuli, the Nrf2 protein will depart from Keap1, resulting in Nrf2 protein stabilization and nuclear translocation, as well as transcriptional activation of antioxidant response (ARE) genes. The majority of the ARE-Nrf2 genes, including *heme oxygenase 1* (*HO1*), *NAD(P)H quinone oxidoreductase-1* (*NQO1*), *glutathione (GSH)*, *γ-glutamyl cysteine ligase catalytic subunit* (*GCLC*), and *modified subunit* (*GCLM*), are antioxidant genes and detoxifying enzymes [10–12].

Activation of Nrf2 cascade, using pharmacological agents, could ameliorate DEX-induced oxidative injury and death in osteoblasts/osteoblastic cells. Li et al. reported that SC79 activated Akt-dependent Nrf2 cascade to inhibit DEX-induced death of murine osteoblasts and MC3T3-E1 osteoblastic cells [13]. Liu et al., have shown that activation of EGFR-Akt-Nrf2 signaling by Icariside II inhibited DEX-induced oxidative injury in MC3T3-E1 cells and murine osteoblasts [14]. Ji et al. found that FGF23 activated Akt downstream Nrf2 signaling to alleviate DEX-induced oxidative injury in osteoblasts [15]. In addition, compound 991 activated AMP-activated protein kinase (AMPK)-dependent Nrf2 signaling cascade, protecting MC3T3-E1 cells and murine osteoblasts from DEX-induced cell death [16]. Zheng et al. showed that activation of Nrf2 signaling by iKeap1, a novel Keap1 inhibitor, ameliorated osteoblast cell apoptosis by DEX and hydrogen peroxide (H_2_O_2_) [17].

MicroRNAs (miRNAs) are small (~22 nucleotides long) and conserved noncoding RNAs (ncRNAs), regulating gene expression by directly binding to the 3′-untranslated regions (3′-UTRs) of target mRNAs [18, 19]. They can lead to translation inhibition and/or degradation of targeted mRNAs [18, 19]. miRNAs can activate the Nrf2 signaling cascade via silencing their target genes [20–22], thereby protecting osteoblasts from DEX. A recent study using the RNAi screening method has discovered the ubiquitin E3 ligase DDB1 and CUL4 associated factor 1 (DCAF1) as a noncanonical regulator of Nrf2 stability and signaling [23]. There is a direct interaction between Nrf2 and DCAF1, the latter can promote Nrf2 ubiquitination and degradation [23]. In the present study, we identified a novel DCAF1-targeting miRNA, microRNA-3175 (miR-3175). Our results showed that DCAF1 silencing by miR-3175 activated Nrf2 signaling to ameliorate DEX-induced oxidative injury and apoptosis in human osteoblasts.

## Materials and methods

### Chemicals, antibodies, and reagents

DEX was obtained from Sigma-Aldrich (St. Louis, MO). All cell culture reagents, including fetal bovine serum (FBS), DMEM, and antibiotics, were provided by Gibco Co. (Gibco; Waltham, MA). Antibodies for HO1 (#70081), NQO1 (#3187), Nrf2 (#12721), Keap1 (#8047), α-Tubulin (#2125), and Lamin B1 (#13435), the cleaved-poly (ADP-ribose) polymerase (PARP, #5625) and the cleaved-caspase-3 (#9664) were provided by Cell Signaling Tech (Shanghai, China). The anti-GCLC antibody (ab55435) was purchased from Abcam (Shanghai, China). From Shanghai Genechem Co. (Shanghai, China), the viral constructs, primers, and plasmids were obtained.

### Cell culture

The primary human osteoblasts were provided by Dr. Ji’s group at Nanjing Medical University [20, 21]. The human osteoblasts were differentiated and cultured as previously described [20, 21], and were used at passage-3 to passage-12. The hFOB1.19 osteoblastic cells were provided by Dr. Ji as well and were cultured in an FBS-containing DMEM medium. Mycoplasma/microbial contamination examination was routinely performed. STR profiling, population doubling time, and morphology were monitored regularly to verify the genotype. The protocols of the current study were approved by the Ethics Committee of Hunan Provincial People’s Hospital, according to the principles of the Declaration of Helsinki.

### Quantitative real-time polymerase chain reaction (qRT-PCR)

TRIzol reagents were added to extract total RNA, which was reverse-transcribed to cDNA. qRT-PCR assays were carried out through an SYBR Green PCR kit (Applied Biosystems, Shanghai, China) under the ABI-7900HT Real-Time PCR System (Applied Biosystems). A 2^-ΔΔ^Ct method was utilized for data quantification. *GAPDH* was always examined as the internal control and the reference gene. For detection of miR-3175 expression, a PrimeScript miRNA qRT-PCR Kit (Takara, Tokyo, Japan) was utilized, with *U6* RNA examined as the internal control. The mRNA primers for the Keap1-Nrf2 cascade genes were provided by Dr. Liu at Jiangsu University [24]. Other verified and specific primers were synthesized by Genechem (Shanghai, China).

### Forced overexpression or inhibition of miR-3175

The miR-3175 precursor sequence (“pre-miR-3175”, Genechem) or the anti-sense sequence (“antagomR-3175”) was inserted into a GV-369 lentiviral construct (Genechem). The construct, along with the lentivirus Helper plasmids (psPAX2 and pMD2.G), were co-transfected to HEK-293T cells. Virus in culture supernatants were enriched and filtered and was added to the primary human osteoblasts or hFOB1.19 osteoblastic cells (cultured in polybrene-containing medium). Puromycin was added to selected stable cells. Expression of mature miR-3175 in stable cells was verified by qRT-PCR assays. The lentiviral microRNA control construct (“Vec”) or the microRNA anti-sense control (“antaC”) were utilized as controls.

### RNA pull-down

A Pierce Magnetic RNA pull-down Kit [25, 26] was utilized for RNA pull-down assays. In short, the biotinylated miR-3175 mimic (Genechem) or control mimic (Genechem) were transfected to primary human osteoblasts for 24 h by Lipofectamine 3000 (Invitrogen, Shanghai, China). Cell lysates (600 μg protein lysates per treatment) were incubated with streptavidin-coated magnetic beads to pull down the biotin-captured RNA complex [25]. Expression of miR-3175-associated *DCAF1 mRNA* was tested by qRT-PCR assay, with its level normalized to the “Input” control.

### RNA fluorescent in situ hybridization (FISH)

Fluorescent in situ hybridization (FISH) kit (RiboBio, Guangzhou, China) was employed for RNA-FISH experiments. Briefly, the FITC (green fluorescence)-labeled miR-3175 probe and the Cy3 (red fluorescence)-labeled *DCAF1* mRNA probe were cotransduced to primary human osteoblasts (at 37 °C for 48 h). Cells were rinsed and observed under a fluorescence microscope (Leica, Shanghai, China).

### RNA-immunoprecipitation (RNA-IP)

Lysates from the primary human osteoblasts (800 μg lysates per treatment) were precleared and incubated with magnetic beads conjugated with the anti-Argonaute 2 antibody (anti-Ago2, Santa Cruz Biotech). After 12 h, beads were washed and incubated with Proteinase K. Thereafter, qRT-PCR was performed to test the purified RNAs (including miR-3175 and *DCAF1* mRNA), and their levels normalized to the “Input” control.

### ARE luciferase reporter assay

Primary human osteoblasts or hFOB1.19 osteoblastic cells were seeded into six-well plates at 60–70% confluence and were transfected with the ARE-inducible firefly luciferase vector (from Dr. Jiang at Nanjing Medical University [27]). Cells were subjected to applied genetic modifications, and cell lysates were examined with the ARE luciferase reporter assay under a luminescence machine [27].

### DCAF1 3′-UTR luciferase activity assay

A pGL4.13 (luc2/SV40) construct encoding DCAF1 3′-UTR was synthesized by Genechem (Shanghai, China). The construct was transfected to primary human osteoblasts or hFOB1.19 osteoblastic cells by Lipofectamine 3000 (Invitrogen, Shanghai, China) together with the Renillaluciferase reporter vector and pRL-SV40 (from Dr. Jiang at Nanjing Medical University [27]). *DCAF1* 3′-UTR luciferase reporter activity in human osteoblasts with applied genetic modifications was tested by a Promega kit [28].

### UTR-null DCAF1 expression

The GV-369 lentiviral construct encoding the 3′-UTR-null DCAF1 was provided by Genechem (Shanghai, China) and was sequence-verified. The primary human osteoblasts were seeded into six-well plates at 60% confluence and were transduced with construct via Lipofectamine 3000. Stable cells were selected by puromycin. The expression of DCAF1 was verified by a Western blotting assay.

### Western blotting

Briefly, cell lysates were extracted, quantified (30 μg per treatment in each lane), and electro-transferred to 10% SDS-PAGE gels. The lysate proteins were then transferred to PVDF blots. After blocking, the blots were incubated with designated primary and corresponding secondary antibodies. To visualize signaling of antigen-antibody binding, the enhanced chemiluminescence (ECL) reagents (Sigma) were applied. An ImageJ software (downloaded from the NIH website) was used for data quantification.

### Mitochondrial depolarization

With mitochondrial depolarization JC-1, fluorescence dye will aggregate into mitochondria to form green monomers [29]. Following treatment, human osteoblasts or hFOB1.19 osteoblastic cells were stained with JC-1 (15.0 μg/mL, Sigma). After 30 min, cells were washed and JC-1 green fluorescence intensity was examined by a Fluoroskan Ascent fluorescence spectrofluorometer (at 488 nm, Hitachi, Japan).

### Single-stranded DNA (ssDNA) ELISA

Human osteoblasts or hFOB1.19 osteoblastic cells were seeded into 96-well plates (at 4500 cells per well). Following treatment, a ssDNA ELISA kit (Roche Diagnostics, Shanghai, China) was utilized to quantify ssDNA contents. The ssDNA ELISA absorbance was tested at 450 nm in each well.

### Reactive oxygen species (ROS) detection

Human osteoblasts or hFOB1.19 osteoblastic cells were seeded into six-well plates (at 100,000 cells per well). Following treatment, cells were stained with 10 μM of CellROX (Invitrogen, Shanghai, China). After 30 min, cells were washed and CellROX red fluorescence intensity was tested under a Fluoroskan Ascent fluorescence spectrofluorometer. CellROX fluorescence images were presented as well.

### Other cell functional assays

The protocols of other cell functional assays, including CCK-8 assaying of cell viability, caspase-3 activity assay, and thiobarbituric acid reactants (TBAR) activity assaying of lipid peroxidation, as well as nuclear TUNEL staining and Annexin V FACS, were described in detail in our previous studies and elsewhere [17, 27, 30, 31].

### Nrf2 silencing

To hFOB1.19 osteoblastic cells, the Nrf2 shRNA lentiviral particles (Santa Cruz Biotech, Santa Cruz, CA) were added. Stable cells were then selected by puromycin. Nrf2 silencing was verified by qRT-PCR assays.

### CRISPR/Cas9-induced gene knockout (KO)

hFOB1.19 cells were seeded into six-well plates at 60% confluence and were transduced with a CRISPR/Cas9-DCAF1-KO-GFP construct (sgRNA targeting: GCCCTGGCATGATGTCTAAT, Genechem, Shanghai, China) or a CRISPR/Cas9-Nrf2-KO-GFP construct (from Dr. Tan [30]). GFP-positive cells were thereby sorted by FACS and selected by a puromycin-containing medium. Cells were distributed to 96-well plates and subjected to DCAF1/Nrf2-KO screening. Single stable cells were then established. Control cells were transduced with the CRISPR/Cas9 PX458-GFP construct with scramble nonsense sgRNA (“koC”).

### Human tissues

As described in ref. [30], human necrotic femoral head tissues and matched surrounding normal femoral head tissues were from a total of 20 (*n* = 20) written-informed consent DEX-treated patients. All patients underwent femoral head resection. miR-3175 and *DCAF1* mRNA expression in fresh tissues was examined by qRT-PCR assays. Protocols were in according to the principles of the Declaration of Helsinki, with approval from the Ethics Committee of Hunan Provincial People’s Hospital.

### Statistical analyses

Data were presented as mean ± standard deviation (SD). For comparison of multiple groups, statistical differences were analyzed by one-way ANOVA (multiple comparisons) with post hoc Bonferroni test (SPSS version 21.0). The unpaired *t*-test (Excel, 2007) was utilized to compare the significance between two treatment groups. *P* < 0.05 was considered statistically significant.

## Results

### microRNA-3175 binds to and silences *DCAF1* in human osteoblasts

**Fig. 1:**
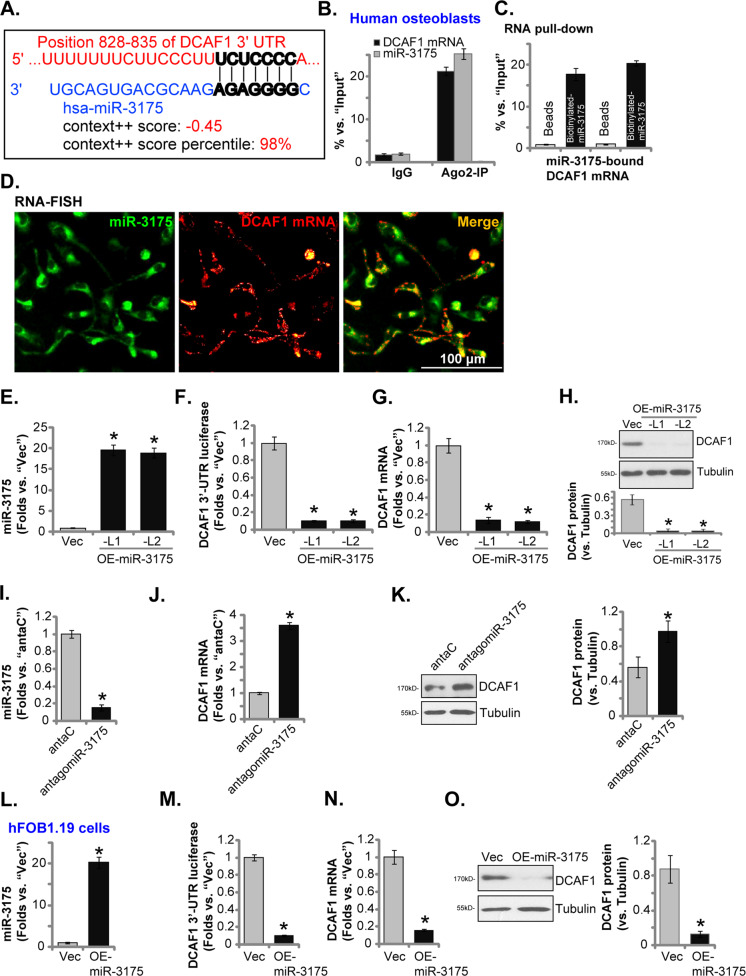
microRNA-3175 binds to and silences DCAF1 in human osteoblasts. TargetScan shows the potential binding sites between miR-3175 and *DCAF1* 3′-UTR (**A**). The lysates of primary human osteoblasts were immunoprecipitated with the Ago2 protein or IgG control, relative expression of miR-3175 and *DCAF1* in the IP lysates was shown (vs. “Input” controls, **B**). The biotinylated miR-3175 mimic or control mimic were transfected to primary human osteoblasts for 24 h. Cell lysates were incubated with streptavidin-coated magnetic beads. Expression of miR-3175-associated *DCAF1 mRNA* was tested by qRT-PCR (vs. “Input” controls, **C**). The FITC-labeled miR-3175 probe and the Cy3-labeled *DCAF1* mRNA probe were cotransduced to primary human osteoblasts, and fluorescence was detected under a fluorescence microscope (**D**). The primary human osteoblasts (**E**–**H**) or hFOB1.19 osteoblastic cells (**L**–**O**), stably expressing a lentiviral construct encoding the miR-3175 precursor (“OE-miR-3175”) or empty vector (“Vec”), were established; Expression of miR-3175 (**E** and **L**), *DCAF1* mRNA (**G** and **N**) and listed proteins (**H** and **O**) were tested by qRT-PCR and Western blotting assays, with *DCAF1* 3′-UTR luciferase activity examined as well (**F** and **M**). Expression of miR-3175 (**I**), *DCAF1* mRNA (**J**), and listed proteins (**K**) in stable human osteoblasts expressing a lentiviral construct encoding miR-3175’s anti-sense (“antagomiR-3175”) or control construct (“antaC”) was shown. Data were presented as mean ± standard deviation (SD, *n* = 5). *P < 0.05 versus “Vec”/“antaC” cells. Experiments were repeated five times, with similar results obtained. Scale bar = 100 μm (**D**).

miRNA binds through complementary base pairing to the 3′-UTR of target mRNA, causing its translational blockage and/or degradation [32, 33]. Computational prediction tools provide a rapid method to identify the putative miRNAs that could bind directly to target mRNA [34]. The miRNA database TargetScan (V7.2) was consulted to explore possible miRNAs targeting 3′-UTR of *DCAF1*. Other miRNA databases, including miRBase, miRNAmap, and miRTarbase, were searched as well to verify the retrieved miRNAs. Three candidate *DCAF1*-targeting miRNAs were retrieved, miR-876-3p, miR-3175, and miR-1236-3p. The context score percentage was >98% and the context^++^ score was < −0.4 [34]. Each of the three miRNA mimics was individually transfected to primary human osteoblasts, their efficiency on *DCAF1* silencing was examined. Bioinformatics studies and preexperimental results identified that miR-3175 potentially targets *DCAF1*’s 3′-UTR (at position 828–835) (Fig. 1A). The binding context score percentage of miR-3175-*DCAF1* 3′-UTR is 98% and the context^++^ score at −0.45 (TargetScan V7.2 [34], Fig. 1A) [34].

Argonaute 2 (Ago2) is a primary component of RNA-induced silencing complex (RISC) that facilitates miRNA binding to its target mRNA. It also promotes the cleavage of the target mRNAs by its endonuclease activity [32, 33]. Ago2 RNA immunoprecipitation (RNA-IP) and subsequent expression analysis of Ago2-immunoprecipitated RNA allows to identify mRNA transcripts enriched in the IP fraction as miRNA targets [32, 33]. As shown in Fig. 1B, the endogenous *DCAF1* mRNA and miR-3175 both co-immunoprecipitated with the anti-Ago2 antibody in human osteoblasts. The nonspecific anti-IgG antibody did not associate *DCAF1* mRNA and miR-3175 (Fig. 1B). The RNA pull-down assay results, Fig. 1C, demonstrated that the biotinylated miR-3175 could pull down endogenous *DCAF1* mRNA in human osteoblasts (Fig. 1C). Moreover, RNA-FISH results showed that miR-3175 (in green fluorescence) co-localized with *DCAF1* mRNA (in red fluorescence) mainly in the cytosol of human osteoblasts (Fig. 1D). Thus, RNA-IP, RNA pull-down, and RNA-FISH assays implied that miR-3175 can directly bind to *DCAF1* mRNA in primary human osteoblasts.

To examine whether miR-3175 could affect *DCAF1* expression, a lentiviral construct encoding the miR-3175 precursor was transduced to human osteoblasts. Via selection by puromycin, two stable lines, OE-miR-3175-L1 and OE-miR-3175-L2, were established. qRT-PCR assays results, Fig. 1E, demonstrated that mature miR-3175 levels increased over 20-folds in OE-miR-3175 osteoblasts (versus control osteoblasts with the empty vector/“Vec”). Dual-luciferase reporter assay results in Fig. 1F demonstrated that forced miR-3175 overexpression robustly decreased *DCAF1* 3′-UTR luciferase activity in human osteoblasts. Furthermore, *DCAF1* mRNA levels were significantly decreased (Fig. 1G). DCAF1 protein levels were downregulated as well with miR-3175 overexpression (Fig. 1H).

Conversely, stable expression of a lentiviral construct encoding miR-3175’s anti-sense (“antagomiR-3175”) potently decreased mature miR-3175 expression (Fig. 1I). Conversely, it increased *DCAF1* mRNA (Fig. 1J) and protein (Fig. 1K) expression in human osteoblasts. In hFOB1.19 osteoblastic cells, forced overexpression of miR-3175 by the same lentiviral miR-3175 precursor construct (“OE-miR-3175”, Fig. 1L) robustly decreased *DCAF1* 3′-UTR luciferase reported activity (Fig. 1M) and silencing *DCAF1* mRNA (Fig. 1N) and protein (Fig. 1O). Collectively, these results suggest that miR-3175 binds to and silences *DCAF1* in human osteoblasts.

### microRNA-3175 activates Nrf2 signaling in human osteoblasts

**Fig. 2:**
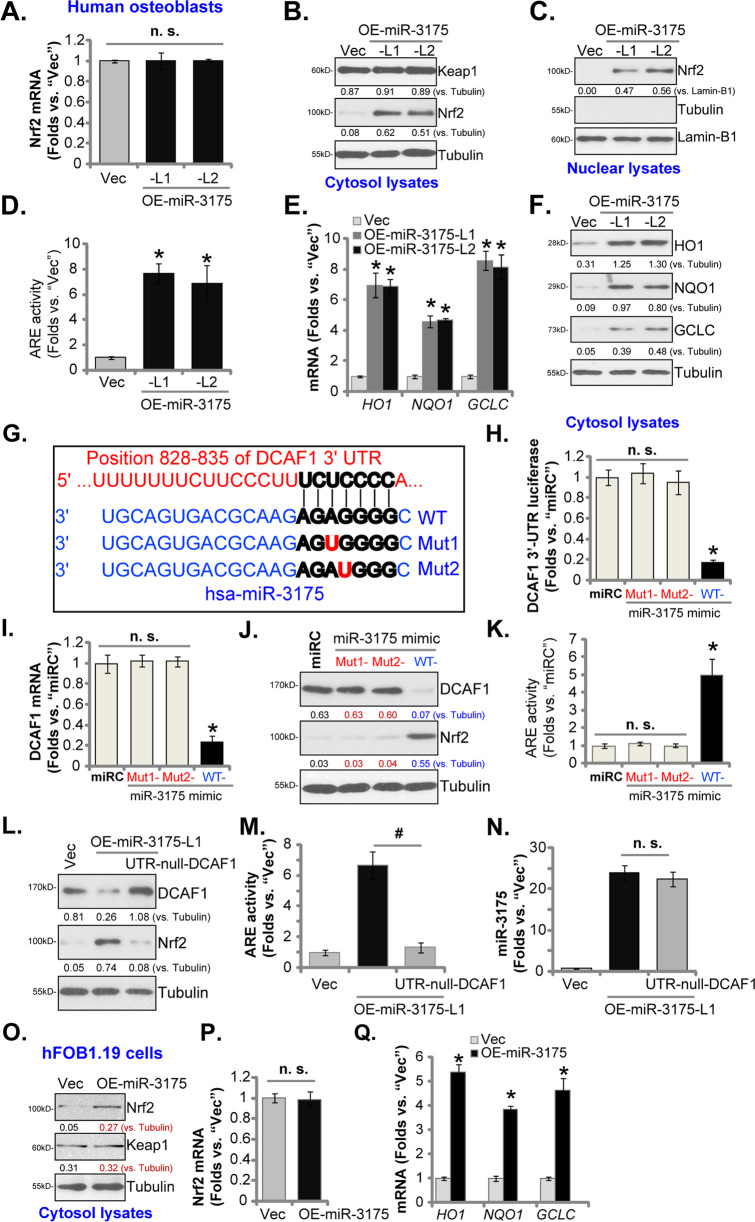
microRNA-3175 activates Nrf2 signaling in human osteoblasts. The primary human osteoblasts were stably transduced with a lentiviral construct encoding the miR-3175 precursor (“OE-miR-3175-L1/L2”, two lines) or the empty vector (“Vec”), listed mRNAs were tested by qRT-PCR assays (**A** and **E**). Expression of the listed proteins in cytosol lysates and nuclear lysates was tested by Western blotting assays (**B**, **C**, and **F**), with the relative ARE activity tested as well (**D**); The primary human osteoblasts were transfected with the applied miR-3175 mimic (wild-type/WT or mutants, listed in **G**) or nonsense control miR mimic (“miR-C”), 500 nM for 48 h, the *DCAF1* 3′-UTR luciferase activity (**H**), *DCAF1* mRNA (**I**), and listed proteins (**J**) expression were tested, with the relative ARE activity examined as well (**K**). OE-miR-3175-L1 human osteoblasts were further transduced with or without a UTR-depleted DCAF1 expression construct (“UTR-null-DCAF1”), control human osteoblasts were with the empty vector (“Vec”); Expression of listed proteins was shown (**L**); The relative ARE activity (**M**) and miR-3175 expression levels (**N**) were presented. The hFOB1.19 osteoblastic cells, stably expressing a lentiviral construct encoding the miR-3175 precursor (“OE-miR-3175”) or empty vector (“Vec”), were established; Expression of listed proteins (**O**) and mRNAs (**P** and **Q**) were tested by Western blotting and qRT-PCR assays, respectively. The expression of listed proteins was quantified and normalized to the loading control. Data were presented as mean ± standard deviation (SD, *n* = 5). *P < 0.05 versus “Vec” cells (**D**, **E**, and **Q**); *P < 0.05 versus “miR-C” cells (**H**, **I**, and **K**); ^#^*P* < 0.05 (**M**); “n. s.” stands for non-statistical difference (**A**, **H**, **K**, **N**, and **P**). Experiments were repeated five times, with similar results obtained.

DCAF1 is a novel E3 ligase required for Nrf2 protein degradation [23]. Since miR-3175 silenced DCAF1, we then tested its effect on Nrf2 signaling. As shown in the miR-3175-overexpressed human osteoblasts, OE-miR-3175-L1 and OE-miR-3175-L2 (see Fig. 1), *Nrf2* mRNA expression was unchanged (Fig. 2A). Western blotting assay results in Fig. 2B, however, demonstrated that Nrf2 protein levels were significantly increased, Keap1 protein expression was however unchanged (Fig. 2B). Testing nuclear fraction proteins, we found that stabilized Nrf2 protein translocated to the nuclei of human osteoblasts after miR-3175 overexpression (Fig. 2C). The ARE activity was significantly increased (Fig. 2D). To further confirm Nrf2 cascade activation, we showed mRNA expression of Nrf2-ARE-dependent genes, including *HO1*, *NQO1*, and *GCLC*, was significantly increased in OE-miR-3175-L1 and OE-miR-3175-L2 osteoblasts (Fig. 2E). Moreover, protein levels of Nrf2-dependent genes were increased (Fig. 2F). These results showed that miR-3175 overexpression activated Nrf2 signaling in human osteoblasts.

To support that DCAF1 silencing is the primary mechanism of Nrf2 cascade activation by miR-3175, we generated two mutant miR-3175 mimics containing mutations at the binding sites to *DCAF1* 3′-UTR (Fig. 2G). The wild-type (“WT”) and the two mutants (“Mut1” and “Mut2”, Fig. 2G) were individually transfected to primary human osteoblasts. As shown WT miR-3175 mimic resulted in significantly decreased *DCAF1* 3′-UTR luciferase activity (Fig. 2H) as well as *DCAF* mRNA (Fig. 2I) and protein (Fig. 2J) expression. While the two mutant mimics were completely ineffective (Fig. 2H–J). Furthermore, the WT miR-3175 mimic, but not the mutants, resulted in Nrf2 protein stabilization (Fig. 2J) and ARE activity increase (Fig. 2K) in human osteoblasts.

To further support our hypothesis, in OE-miR-3175-L1 human osteoblasts, a UTR-depleted DCAF1 expression construct (“UTR-null-DCAF1”) was transduced. The construct restored DCAF1 protein expression in miR-3175-overexpressed osteoblasts (Fig. 2L). Consequently, miR-3175 overexpression-induced Nrf2 protein stabilization (Fig. 2L) and ARE activity increase (Fig. 2M) were reversed by the UTR-depleted DCAF1. miR-3175 expression was unchanged (Fig. 2N). These results supported that in human osteoblasts miR-3175 overexpression-induced Nrf2 cascade activation by silencing DCAF1.

In hFOB1.19 osteoblastic cells forced overexpression of miR-3175 (“OE-miR-3175”, see Fig. 1) increased Nrf2 protein (Fig. 2O) but not *Nrf2* mRNA (Fig. 2P). Keap1 protein was again unchanged (Fig. 2O). mRNA levels of Nrf2-ARE-dependent genes, *HO1*, *NQO1*, and *GCLC*, were significantly increased in OE-miR-3175-hFOB1.19 cells (Fig. 2Q). Thus, miR-3175 activated Nrf2 signaling cascade in hFOB1.19 cells.

### microRNA-3175 attenuates DEX-induced oxidative injury in human osteoblasts

**Fig. 3:**
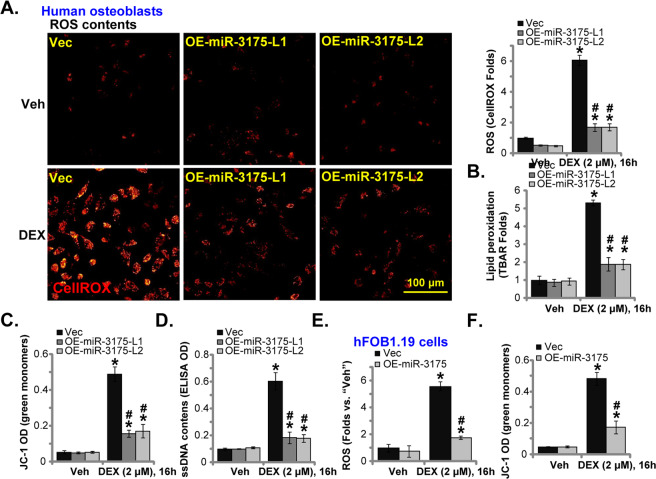
microRNA-3175 attenuates DEX-induced oxidative injury in human osteoblasts. The primary human osteoblasts (**A**–**D**) or hFOB1.19 osteoblastic cells (**E** and **F**), stably expressing the lentiviral construct encoding the miR-3175 precursor (“OE-miR-3175”) or empty vector (“Vec”), were established and treated with dexamethasone (DEX, 2 μM) or vehicle control (“Veh”); Cells were further cultured for 16 h; Cellular ROS contents, lipid peroxidation intensity, mitochondrial depolarization, and DNA damage were tested by CellROX staining (**A** and **E**), TBAR activity (**B**), JC-1 monomers intensity (**C** and **F**), and single-strand DNA (ssDNA) ELISA (**D**) assays, respectively, and results quantified. Data were presented as mean ± standard deviation (SD, *n* = 5). *P < 0.05 versus “Veh” treatment in “Vec” cells. ^#^*P* < 0.05 versus “DEX” treatment in “Vec” cells. Experiments were repeated five times, with similar results obtained. Scale bar = 100 μm (**A**).

Activation of Nrf2 cascade could inhibit DEX-induced oxidative injury and cell death in osteoblasts/osteoblastic cells [13–15, 20]. Since *DCAF1* silencing by miR-3175 activated Nrf2 cascade, we tested its effect on DEX-induced oxidative stress. Testing cellular ROS contents, by CellROX staining assays, showed that DEX stimulation led to significant ROS production in vector control human osteoblasts (Fig. 3A). In miR-3175-overexpressed human osteoblasts, DEX-induced ROS production was however largely attenuated (Fig. 3A). In addition, in vector control human osteoblasts DEX induced significant lipid peroxidation, mitochondrial depolarization, and DNA damage, which were tested by TBAR activity increase (Fig. 3B), JC-1 green monomers formation (Fig. 3C), and single-strand DNA (ssDNA) accumulation (Fig. 3D), respectively. Such actions by DEX were largely ameliorated following ectopic overexpression of miR-3175 (Fig. 3B–D).

In vector control hFOB1.19 osteoblastic cells DEX stimulation-induced ROS production (CellROX intensity increase, Fig. 3E) and mitochondrial depolarization (JC-1 green monomers increase, Fig. 3F). miR-3175 overexpression (OE-miR-3175, see Fig. 1) potently inhibited DEX-induced oxidative stress in hFOB1.19 cells (Fig. 3E, F).

### microRNA-3175 attenuates DEX-induced apoptosis activation in human osteoblasts

**Fig. 4:**
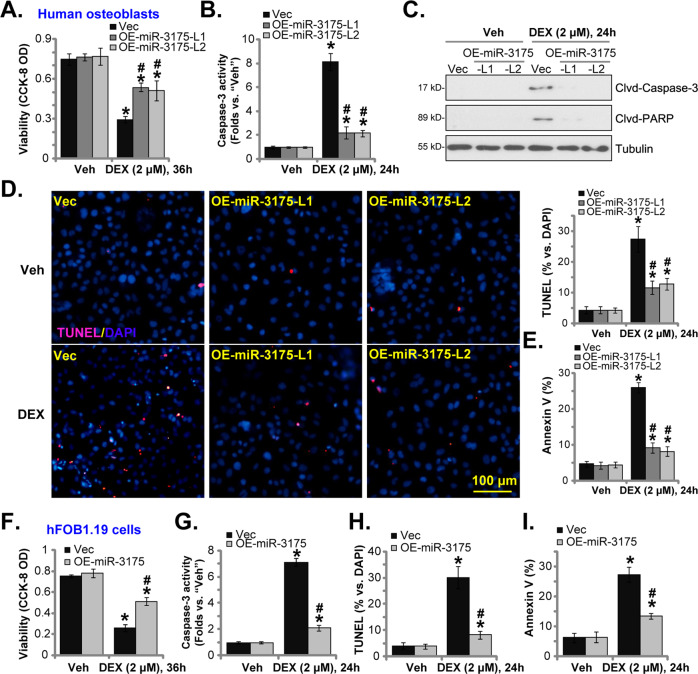
microRNA-3175 attenuates DEX-induced apoptosis activation in human osteoblasts. The primary human osteoblasts (**A**–**E**) or hFOB1.19 osteoblastic cells (**F**–**I**), stably expressing a lentiviral construct encoding the miR-3175 precursor (“OE-miR-3175”) or empty vector (“Vec”), were established and treated with dexamethasone (DEX, 2 μM) or vehicle control (“Veh”); Cells were further cultured for applied time periods; Cell viability was tested by CCK-8 assays (**A** and **F**); The caspase-3 activity (**B** and **G**) and caspase-3-PARP cleavages (**C**) were examined. Cell apoptosis was tested by recording TUNEL-positive nuclei ratio (**D** and **H**) and Annexin V percentage (**E** and **I**), with results quantified. The nuclear TUNEL ratio, % vs. DAPI, from at least 1500 cells in five random views per treatment was calculated. Data were presented as mean ± standard deviation (SD, *n* = 5). *P < 0.05 versus “Veh” treatment in “Vec” cells. ^#^*P* < 0.05 versus “DEX” treatment in “Vec” cells. Experiments were repeated five times, with similar results obtained. Scale bar = 100 μm (**D**).

Next, we tested the potential effect of miR-3175 on DEX-induced osteoblast cell death. Figure 4A demonstrated that treatment with DEX (2 μM, 36 h) potently decreased the number of viable vector control human osteoblasts, which was largely inhibited after miR-3175 overexpression (Fig. 4A). Furthermore, in control osteoblasts, DEX stimulation led to caspase-3 activity increase (Fig. 4B) as well as cleavages of caspase-3 and PARP (Fig. 4C), which were largely attenuated by miR-3175 overexpression (Fig. 4B, C). Significant apoptosis activation was detected in DEX-treated vector control human osteoblasts, evidenced by increased TUNEL-positive nuclei ratio (Fig. 4D) and Annexin V positive staining (Fig. 4E). With miR-3175 overexpression, DEX-induced apoptosis activation was largely inhibited (Fig. 4D, E).

Similarly in the vector control hFOB1.19 osteoblastic cells, DEX induced viability reduction (Fig. 4F), caspase-3 activation (Fig. 4G), and apoptosis activation (Fig. 4H, I). With miR-3175 overexpression (“OE-miR-3175”), DEX-induced cytotoxicity (Fig. 4F) and apoptosis activation (Fig. 4G–I) were largely attenuated. Thus miR-3175 overexpression attenuated DEX-induced apoptosis activation in human osteoblasts.

### miR-3175-induced osteoblast cytoprotection against DEX requires activation of Nrf2 signaling

**Fig. 5:**
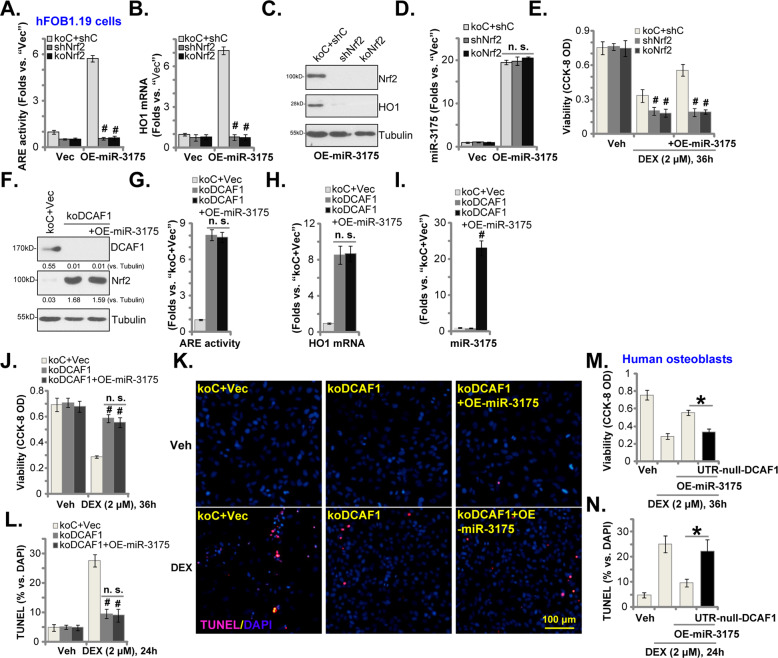
miR-3175-induced osteoblast cytoprotection against DEX requires activation of Nrf2 signaling. The hFOB1.19 osteoblastic cells, stably expressing lentiviral Nrf2 shRNA (“shNrf2”), a CRISPR/Cas9-Nrf2-KO-puro construct (“koNrf2”) or control shRNA plus Cas9-control construct (“koC+shC”), were established, cells were further transduced with a lentiviral construct encoding the miR-3175 precursor (“OE-miR-3175”) or empty vector (“Vec”), the relative ARE activity (**A**) and expression of listed genes (**B**–**D**) were shown. Alternatively, cells were treated with dexamethasone (DEX, 2 μM) or vehicle control (“Veh”) and cultured for applied time periods; Cell viability was tested by CCK-8 assays (**E**). The hFOB1.19 osteoblastic cells, stably expressing a CRISPR/Cas9-DCAF1-KO-puro construct (“ko-DCAF1”), were further transduced with or without a lentiviral construct encoding the miR-3175 precursor (“ko-DCAF1+OE-miR-3175”). Control cells were with miR control vector plus Cas9-control construct (“koC+Vec”); Expression of listed genes (**F**, **H**, and **I**) and the relative ARE activity (**G**) were tested. Cells were also treated with dexamethasone (DEX, 2 μM) or vehicle control (“Veh”) and were cultured for applied time periods; Cell viability (**J**) and apoptosis (**K** and **L**) were tested similarly. The primary human osteoblasts, stably expressing the lentiviral construct encoding the miR-3175 precursor (“OE-miR-3175”) were further transduced with or without a UTR-depleted DCAF1 expression construct (“UTR-null-DCAF1”); Cells were treated with dexamethasone (DEX, 2 μM) or vehicle control (“Veh”) and cultured for applied time periods; Cell viability (**M**) and apoptosis (**N**) were tested similarly. The expression of listed proteins was quantified and normalized to the loading control. The nuclear TUNEL ratio, % vs. DAPI, from at least 1500 cells in five random views per treatment was calculated. Data were presented as mean ± standard deviation (SD, *n* = 5). ^#^*P* < 0.05 versus “koC+shC” cells (**A**–**E**); ^#^*P* < 0.05 versus “koC+Vec” cells (**I**–**L**). *P < 0.05 (**M**). Experiments were repeated five times, with similar results obtained. Scale bar = 100 μm (**K**).

To support that Nrf2 cascade activation is the primary reason of miR-3175-induced osteoblast cytoprotection against DEX, genetic methods were employed to deplete Nrf2. The Nrf2 shRNA lentiviral particles were added to hFOB1.19 cells, and stable cells were established after selection (“shNrf2 cells”). Moreover, the CRISPR/Cas9 gene-editing method was employed to complete knockout Nrf2 in hFOB1.19 cells, and single stable cells were established (“koNrf2 cells”). As shown, miR-3175 overexpression-induced ARE activity increase (Fig. 5A) as well as *HO1* mRNA (Fig. 5B) and protein (Fig. 5C) expression were completely blocked by Nrf2 shRNA or KO in hFOB1.19 cells. Furthermore, Nrf2 protein stabilization was reversed with Nrf2 depletion (Fig. 5C). Unsurprisingly, miR-3175 expression was unaffected by Nrf2 silencing or KO in hFOB1.19 cells (Fig. 5D). DEX-induced viability (CCK-8 OD) reduction (Fig. 5E) was significantly intensified in hFOB1.19 cells with Nrf2 silencing or KO. Importantly, miR-3175 overexpression failed to inhibit DEX-induced cytotoxicity in Nrf2-silenced and Nrf2-KO cells (Fig. 5E). These results showed that Nrf2 silencing or depletion abolished miR-3175-induced osteoblast cytoprotection against DEX.

Next, a CRISPR/Cas9-DCAF1-KO-puro construct was transduced to hFOB1.19 cells. Following selection by puromycin and *DCAF1* KO screening, single stable cells were established: ko-DCAF1 cells. As shown, DCAF1 protein was depleted in ko-DCAF1 hFOB1.19 cells (Fig. 5F). DCAF1 KO activated Nrf2 signaling by inducing Nrf2 protein stabilization (Fig. 5F), ARE activity increase (Fig. 5G), and *HO1* expression (Fig. 5G, H). Ectopic overexpression of miR-3175 (Fig. 5I) in ko-DCAF1 hFOB1.19 cells, however, failed to further increase Nrf2 cascade activation (Fig. 5F–H). Functional studies showed that DCAF1 KO largely inhibited DEX-induced viability (CCK-8 OD) reduction (Fig. 5J) and cell apoptosis (TUNEL-positive nuclei ratio increase, Fig. 5K, L). Importantly, ectopic miR-3175 overexpression failed to offer additional osteoblast cytoprotection against DEX in ko-DCAF1 cells (Fig. 5J–L). These results further supported that miR-3175 overexpression-induced osteoblast cytoprotection against DEX is through silencing DCAF1.

To further support our hypothesis, the UTR-null-DCAF1 construct was transduced to miR-3175-overexpressed primary human osteoblasts. As shown, restoring DCAF1 expression by the UTR-null-DCAF1 (see Fig. 2L) largely attenuated miR-3175 overexpression-induced osteoblast cytoprotection against DEX (Fig. 5M, N). In other words, miR-3175 overexpression was ineffective on DEX-induced viability reduction (Fig. 5M) and apoptosis (Fig. 5N) when DCAF1 expression was rescued by the UTR-null-DCAF1 in human osteoblasts.

### miR-3175 is downregulated in necrotic femoral head tissues of DEX-taking patients

**Fig. 6:**
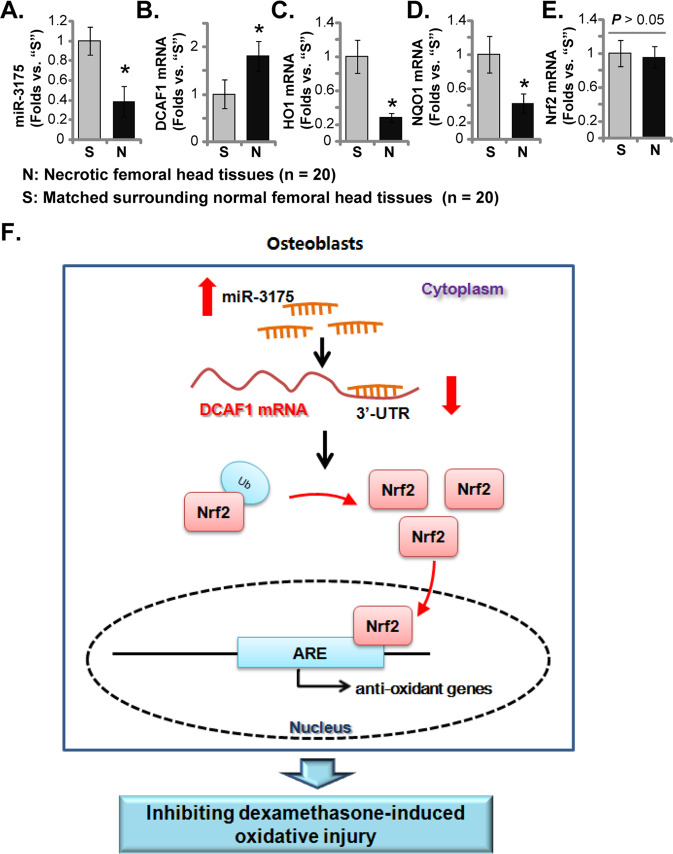
miR-3175 is downregulated in necrotic femoral head tissues of DEX-taking patients. Expression of miR-3175 (**A**) and listed mRNAs (**B**–**E**) in necrotic femoral head tissues (“N”) and surrounding normal femoral head tissues (“S”) of 20 different DEX-taking patients (*n* = 20) was tested by qRT-PCR assays. Data were expressed as mean ± standard deviation (SD). *P < 0.05 versus “S” tissues. The proposed signaling carton of study (**F**).

At last, expression of miR-3175 in necrotic femoral head tissues of DEX-taking human patients was tested by qRT-PCR assays. As shown miR-3175 expression in the necrotic femoral head tissues (“N”) was dramatically lower than that in matched surrounding normal bone tissues (“S”) (Fig. 6A). Conversely, upregulation of *DCAF1* mRNA was detected in necrotic femoral head tissues (*P* < 0.05 versus “S” tissues, Fig. 6B). *HO1* (Fig. 6C), and *NQO1* (Fig. 6D) mRNA levels were significantly downregulated in necrotic femoral head tissues, indicating Nrf2 cascade inhibition. *Nrf2* mRNA expression was however not significantly different between the two groups (Fig. 6E). Therefore, miR-3175 is downregulated in human necrotic femoral head tissues, correlating with *DCAF1* upregulation (Pearson Correction = −0.68), *HO1* (Pearson Correction = −0.79), and *NQO1* (Pearson Correction = 0.69) mRNA downregulation (Nrf2 cascade inhibition).

## Discussion

The function and the potential targets of miR-3175 are still largely unknown. Qi et al. reported that miR-3175 acted as a tumor-suppressive miRNA and was downregulated in human glioma. Transfection of miR-3175 mimics induced proliferation inhibition and apoptosis in glioma cells possibly by inhibiting the PI3K-Akt pathway [35]. Xia et al. found that LncRNA LINC00520 sponged miR-3175 to promote lung cancer cell growth [36]. Han et al. however reported cancer-promoting activity of miR-3175, as it silenced the tumor suppressor HOXB1 in human glioma [37]. miR-3175 downregulation inhibited cell proliferation and invasion and promoted apoptosis in glioma cells [37]. Zhong et al., also reported that miR-3175 silenced Smad7 to promote epithelial-mesenchymal transition (EMT) in human conjunctiva and pterygium [38].

The results of the present study indicated that miR-3175 is a novel *DCAF1*-targeting miRNA in human osteoblasts. RNA pull-down, Ago2 RNA-IP, and RNA-FISH experiment results all supported a direct binding between miR-3175 and *DCAF1* mRNA in primary human osteoblasts. In human osteoblasts and hFOB1.19 osteoblastic cells, forced overexpression of miR-3175 inhibited *DCAF1* 3′-UTR luciferase activity and downregulated *DCAF1* expression. Conversely, miR-3175 silencing by antagomiR-3175 increased *DCAF1* expression. Importantly, the mutant miR-3175 mimics, containing mutations at the proposed binding sites to *DCAF1* 3′-UTR, failed to alter *DCAF1* expression in human osteoblasts.

We further showed that silencing of DCAF1 by miR-3175 activated Nrf2 signaling in human osteoblasts and hFOB1.19 cells, causing Nrf2 protein stabilization, ARE activity increase, transcriptional activation of Nrf2-dependent genes (*HO1*, *NQO1*, and *GCLC*). Yet transfection of the two mutant miR-3175 mimics failed to activate Nrf2 signaling in human osteoblasts. Moreover, restoring DCAF1 expression, by the UTR-null-DCAF1 construct, abolished miR-3175 overexpression-induced Nrf2 cascade activation in human osteoblasts. These results supported that DCAF1 silencing is the key mechanism responsible for Nrf2 cascade activation by miR-3175 (Fig. 6F).

Different miRNAs could protect osteoblasts/osteoblastic cells from DEX-induced oxidative injury and cell death. For example, Zhao et al. reported that Keap1 silencing by miR-200a activated Nrf2 signaling to protect osteoblastic cells from DEX [20]. In human osteoblasts miR-19a silenced tuberous sclerosis complex 1 (TSC1) to activate the mTORC1-dependent Nrf2 signaling cascade, protecting human osteoblasts from DEX-induced oxidative injury and cell death [21]. Inhibition of miR-107, a CAB39 (calcium-binding protein 39)-targeting microRNA, activated AMPK-dependent Nrf2 signaling cascade to protect human osteoblasts from DEX [22].

We found that activation of Nrf2 signaling by miR-3175 potently inhibited DEX-induced oxidative injury in human osteoblasts. DEX-induced ROS production, lipid peroxidation, mitochondrial depolarization, and DNA damage were all largely attenuated in miR-3175-overexpressed human osteoblasts. Furthermore, forced overexpression of miR-3175 largely inhibited DEX-induced cytotoxicity and apoptosis in human osteoblasts. Importantly, we found that activation of the Nrf2 cascade was required for miR-3175-induced osteoblast cytoprotection against DEX. In hFOB1.19 cells, Nrf2 shRNA or KO abolished miR-3175 overexpression-induced osteoblast cytoprotection. Conversely, DFAC1 KO activated the Nrf2 cascade and inhibited DEX-induced cytotoxicity in hFOB1.19 cells. Significantly, miR-3175 overexpression failed to further increase Nrf2 cascade activation nor inhibiting DEX-induced cytotoxicity in DCAF1 KO hFOB1.19 cells. These results clearly demonstrated that DCAF1 silencing by miR-3175 activated Nrf2 signaling to inhibit DEX-induced oxidative injury and death in human osteoblasts (Fig. 6E).

## Conclusion

miR-3175 is a novel DCAF1-targeting miRNA. Silencing DCAF1 by miR-3175 activated Nrf2 signaling to inhibit DEX-induced oxidative injury and apoptosis in human osteoblasts.

## Data Availability

The data are included in the article.
